# Long-Term Diabetes Improvement After Duodenal Exclusion in Zucker Diabetic Fatty Rats Is Associated with Prevention of Strain-Specific Pancreatic Remodeling and Increased Beta Cell Proliferation

**DOI:** 10.1007/s11695-022-06040-w

**Published:** 2022-04-06

**Authors:** Gabriel Seifert, Ambrus Malyi, Peter Bronsert, Sven Plohmann, Rebeccca Kesselring, Stefan Fichtner-Feigl, Goran Marjanovic, Jodok Matthias Fink, Claudia Laessle

**Affiliations:** 1 Department of General and Visceral Surgery, Medical Center, Faculty of Medicine, University of Freiburg, University of Freiburg, Hugstetter Straße 55, 79106 Freiburg, Germany; 2 Institute of Translational Medicine, Semmelweis University, Budapest, Hungary; 3 Institute for Surgical Pathology, Medical Center - University of Freiburg, Faculty of Medicine, University of Freiburg, Breisacher Straße 115A, 79106 Freiburg, Germany; 4 Core Facility Histopathology and Digital Pathology, Faculty of Medicine, Medical Center, University of Freiburg, Breisacher Straße 115A, 79106 Freiburg, Germany

**Keywords:** Metabolic surgery, Duodeno-jejunal bypass, Diabetes, Pancreatic, Islet cells, Zucker rat, Diabetes surgery

## Abstract

**Background:**

Response to metabolic surgery is heterogeneous and the metabolic states that underpin weight loss and metabolic improvement are still unclear. In this study, we investigate parameters of post-bariatric fasting glucoregulation and leverage artificial intelligence-assisted whole-slide image analyses to characterize associated immunohistologic features of the pancreas.

**Materials and Methods:**

We performed either loop duodeno-jejunostomy (DJOS) with exclusion of 1/3 of total intestinal length, loop duodeno-ileostomy with exclusion of 2/3 of total intestinal length (DiOS), or a sham operation on 8-week-old male obese ZDF rats. Six months post-operative, we measured blood metabolites and hormones. Subsequently, pancreatic and intestinal tissue was removed, formalin fixed, and paraffin embedded. Immunohistologic (IHC) analyses included proliferating cell nuclear antigen (PCNA) to visualize the proliferation fraction and pancreatic and duodenal homeobox 1 (PDX 1) as a measure of pancreatic cell differentiation. For IHC quantification, all slides were digitalized and analyzed using QuPath. All analyzed slides were reviewed by two independent pathologists for correctness.

**Results:**

DJOS and DiOS were associated with preserved fasting insulin production compared to sham. Histopathologic evaluation showed significantly higher numbers of beta cells and specifically of clustered cell organization in DJOS and DiOS compared to sham. Cell proliferation (PCNA) was significantly elevated in DJOS and DiOS compared to sham.

**Conclusion:**

In this interventional model of bariatric surgery in severe genetic diabetes, we demonstrate post-operative histologic and immunohistologic features of the pancreas associated with improved fasting glucose homeostasis.

**Graphical abstract:**

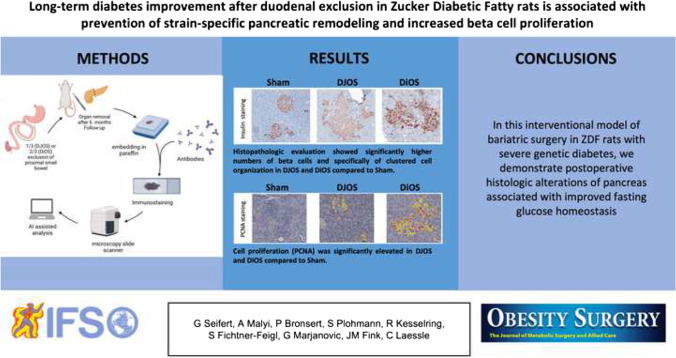

## Introduction

Obesity is associated with significant risk of developing type 2 diabetes (T2D) [1]. In mild and moderate forms of obesity-related T2D, metabolic surgery is associated with partial to complete remission, even in the long term [2–6]. Several guidelines have integrated metabolic surgery into diabetes therapy algorithms [2, 7]. Roux-Y gastric bypass (RYGB) is the most frequently performed surgery in this indication. Obesity-related diabetes is characterized by peripheral insulin resistance and progressive loss of beta cells [8–10]. Dedifferentiation and apoptosis of beta cells are associated with persistent hyperglycemia, increased inflammation, cytokines, and free fatty acids [11–14]. The molecular short- and long-term mechanisms of metabolic surgery that lead to improved glucoregulatory control are complex and in part weight loss independent [15, 16]. Improved insulin sensitivity and secretion are associated with multiple metabolic, hormonal, and microbiome changes [17–19]. A recent study reports re-differentiation of beta cells and weight-independent diabetes improvement following metabolic surgery [20].

The natural course of ZDF rats, which are characterized by a leptin receptor mutation, is associated with changes in islet morphology, severe insulin resistance, and diabetes progression [21]. In a previous study, we tested the hypothesis that common channel length after duodenal exclusion is associated with T2D remission [22]. Common channel length was not associated with improved diabetes remission, but short common channel length was associated with improved weight loss and increased peripheral insulin sensitivity. In this study, we examine immunohistologic features of morphology, differentiation, and proliferation of pancreatic beta cells associated with a shorter or longer common channel after duodenal exclusion in a ZDF rat model. To reduce examiner bias, we used an artificial intelligence (AI)-assisted quantitative approach. To our knowledge, comparable data on these late post-operative changes in ZDF rats have not been published so far.

## Material and Methods

### Diets and Animals

Zucker diabetic fatty rats (ZDF—*Lepr*^*fa*^/Crl) were obtained from Charles River Breeding Laboratories (Wilmington, MA). Animals were male and 7 weeks old at onset of the experiment. Animal care and feeding protocol were conducted as described previously [22]. Four hours before surgery, rats were put on a fasting regimen. For hormone and blood metabolite measurement, the time interval was extended to 6-h fasting. Experimental protocols were authorized by the local animal welfare committee. All applicable institutional and national guidelines for the care and use of animals were followed.

### Experimental Protocols

Rats were purchased and acclimatized for 14 days with free access to food and water. For evaluation of hormone and metabolite levels, 400 µl venous blood were drawn via cannulization of one of the tail veins. After euthanization 6 months post-surgery, pancreas, terminal ileum, duodenum, biliopancreatic limb, and ascending colon were collected for immunohistochemical staining.

Surgical procedures and blood parameter measurements have been previously described and will be summarized below for clarity [22].

### Surgery

**Fig. 1:**
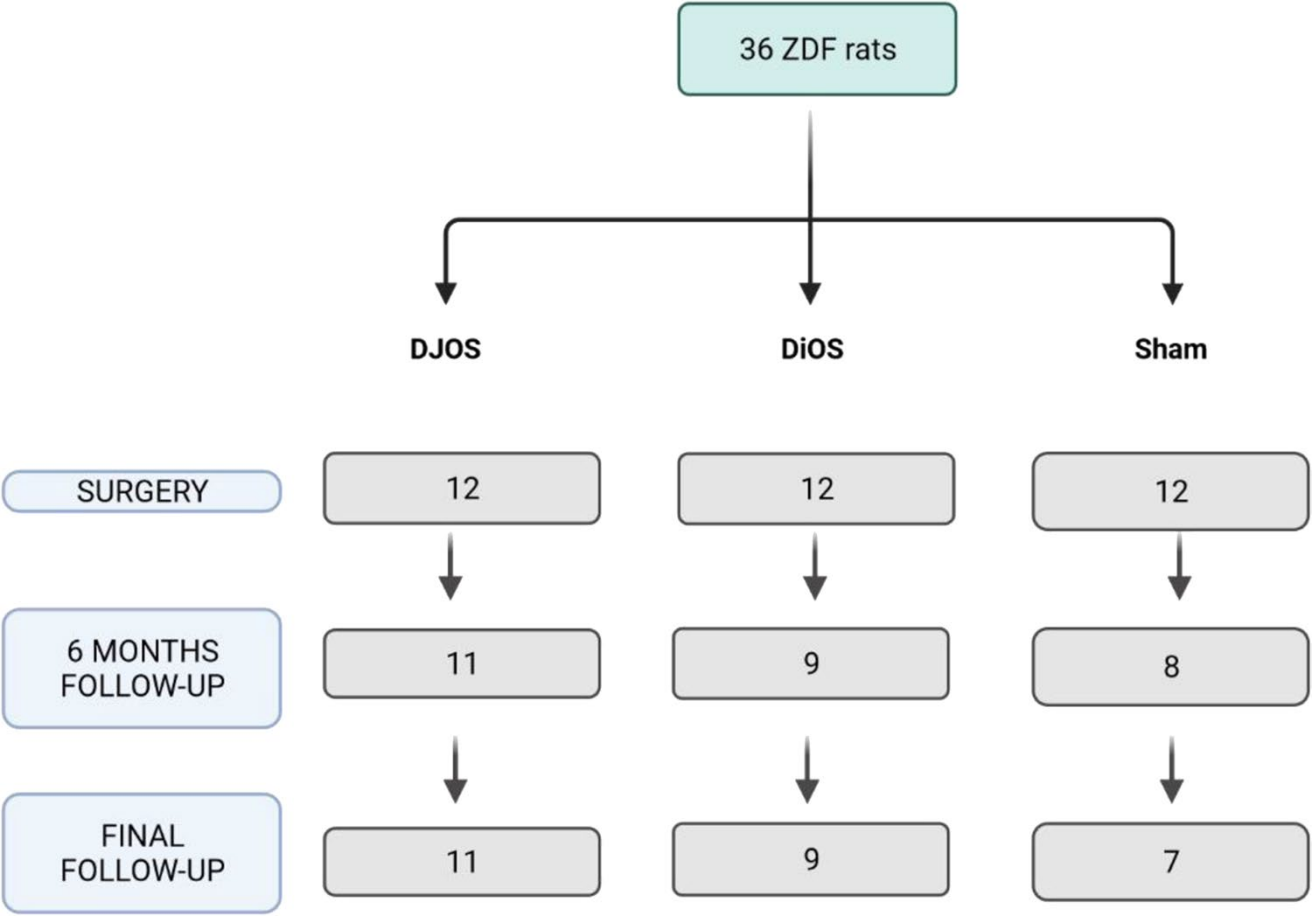
Flowchart of timeline and mortality

After opening the abdomen in the midline of 3–4 cm, the length of the small intestine was measured. Afterwards, the duodenum was transected distally of the pylorus. The distal duodenal stump was closed with 3–4 single stitches, using PDS 6/0 (Ethicon). At the pre-defined position, an antecolic end-to-side duodeno-jejunostomy (DJOS) or duodeno-ileostomy (DiOS) was performed using single stitches. The mesenteric space was closed using PDS 6/0 (Ethicon). In summary, DJOS and DiOS both surgically bypass the duodenum as well as either ~ 1/3 (DJOS) or ~ 2/3 (DiOS) of total small intestinal length. After surgery, animals were housed alone and with free access to water as well as liquid high caloric food (Fresubin energy drink, Fresenius Kabi Deutschland GmbH, Bad Homburg, Germany). Oral food was continuously increased until 5 days after operation. The overall mortality rate at 6-month follow-up was 25% (Fig. 1).

### Hormone and Metabolite Measurement

For the current investigation, we analyzed hormone levels after 6 h of fasting and collected 400 µl blood via cannulization of the tail vein, using tubes containing 0.69 mg K3EDTA (Sarstedt AG & Co, Nümbrecht, Germany).

High-range rat insulin ELISA was a solid phase two-site enzyme immunoassay using HRP reaction for detection (DRG Instruments GmbH, Marburg, Germany). Glucagon-like peptide 1 (GLP-1) (7–36) samples were added directly to a straptavidin-coated microtiter plate (EMD Millipore Corporation, Darmstadt, Germany). High-sensitive CRP was measured using Human C-Reactive Protein/CRP DuoSet ELISA kit (DY1707, R&D Systems Inc., Minneapolis, USA). Free fatty acids were measured using Free Fatty Acid Quantification Kit from Abcam (#ab65341, Abcam, Cambridge, UK)

### Immunohistochemistry

Tissue of the pancreatic corpus and tail as well as intestine were fixed in 4% paraformaldehyde solution and embedded in paraffin. Sections of 3 μm were harvested to analyze pancreatic parenchyma, specifically beta cells and L cells in the duodenum, alimentary limb, terminal ileum, and ascending colon. Tissue samples were stained for insulin, PDX 1, PCNA, GLP-1, and HE.

For GLP-1 staining, sections were deparaffinized in Rotihistol (Roth, Karlsruhe, Germany) and subjected to antigen retrieval in 1 mM Tris/1 mM EDTA pH 9. Sections were blocked for endogenous peroxidase activity and non-specific binding with 5% goat serum followed by incubation with primary rat anti-GLP-1 antibody (1∶5000, GLP1-#22,625, Abcam, Cambridge, UK). Sections were visualized using Dako Envision (K4003-HRP, Dako North America, Carpinteria, CA) and developed using 3-amino-9-ethylcarbazol as a chromagen before they were dehydrated with Rotihistol (Roth, Karlsruhe, Germany) and preserved with Roti-Histokitt (Roth, Karlsruhe, Germany).

For PDX-1 and PCNA staining, sections were deparaffinized in Rotihistol (Roth, Karlsruhe, Germany) and subjected to antigen retrieval in 10 mM citrate buffer, pH 6.0 (1.92 g citric acid monohydrate/1 L dH_2_0 pH 6.0 with NaOH; Merck; #244.1000). Sections were blocked for endogenous peroxidase activity and non-specific binding with Peroxidase-Blocking Solution (DAKO #S2023) followed by incubation with primary antibody (PDX: 1:500, #219,207, Abcam, Cambridge, UK, PCNA 1:30,000 #29, Abcam, Cambridge, UK). Sections were visualized using Dako Envision (K4003-HRP, Dako North America, Carpinteria, CA) and developed using DAB + Substrate Chromogen System (#K3468; DAKO) before they were dehydrated with Rotihistol (Roth, Karlsruhe, Germany) and preserved with Roti-Histokitt (Roth, Karlsruhe, Germany). For insulin staining, Sects. (4 µm) were deparaffinized, rehydrated, and pre-treated in a pressure cooker (or in a microwave) in a Tris–EDTA method pH 9.0. They were then incubated with a monoclonal rabbit primary antibody anti-insulin 1:64,000 (0.950 mg/ml; #ab181547; Abcam). Slides were washed with TBS/0.05% Tween20 and then developed with EnVision-System (DAKO) according to the manufacturer’s protocol.

#### Digital Assessment

We digitized conventional stains (H&E) and immunohistochemical (IHC) utilizing a VENTANA DP 200 slide scanner (Roche Diagnostics, Rotkreuz, Switzerland). Digitalization was carried out on a single focus layer at 20-fold magnification. All digitized slides were reviewed by two pathologists and one surgeon and subsequently analyzed using QuPath (QuPath version 0.2.3, created at the Queens University of Belfast, Northern Ireland).

Mounting the smart annotation toolkit of QuPath, every isle of Langerhans was allocated. Positive and negative beta cells were sorted according to the mean DAB optical density of their nucleus. Annotation of the Langerhans islets and the sorting of the IHC positive or negative cells were conducted via QuPath [23]. Quantitative measurements were performed to obtain the absolute and relative number of positive and negative cellular detection. For histologic evaluation of the pancreatic islets in the corpus and tail area, the number and morphologic appearance of five randomly selected vision fields, each with fivefold magnification, were determined. Beta cell scoring was performed as follows. Large islet cell clusters (defined as more than 10 beta-cells in a cluster) were weighted twofold, and isolated islet cells were weighted once and added together. For histological scoring of L cells, the number of GLP-1 positive cells in randomly selected cross-sections of duodenum, alimentary limb, terminal ileum, and ascending colon adding up to 3000 μm luminal length in each defined segment was counted.

### Statistical Analysis

Statistical analyses were conducted using Prism 9 for Mac Version 9.2.0 (GraphPad Software Inc.). A Mann–Whitney test was used to compare single values. *P* values of < 0.05 were considered as significant. Visual graphics were created with BioRender.com.

## Results

DJOS and DiOS were significantly associated with improved GLP-1, insulin and lipid metabolism, beta cell protection, a higher count of intestinal L cells, and beta cell markers of differentiation and proliferation.

### Serum Insulin, Glucagon-Like Peptide 1, and Free Fatty Acids

**Fig. 2:**
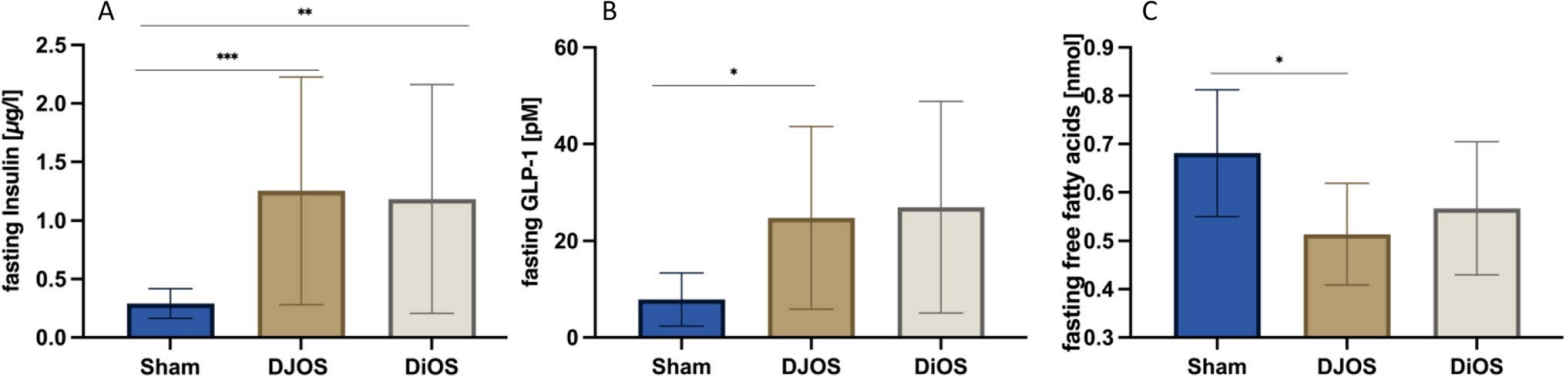
Box plot of serum hormone and metabolite levels. **A** Fasting insulin levels 6 months after surgery with highly limited insulin secretion in sham group (Mann–Whitney test, sham vs DJOS *p* = 0.0002 and DiOS vs sham *p* = 0.0035). **B** Fasting GLP-1 levels 6 months after surgery with a significant increase of GLP-1 in DJOS (Mann–Whitney test, sham vs DJOS *p* = 0.0136). **C** Lower free fatty acids in DJOS and DiOS 6 months after operation (Mann–Whitney test, sham vs DJOS *p* = 0.0295)

DJOS and DiOS were associated with significantly higher basal serum insulin levels compared to sham 6 months post-operatively. Sham had dramatically reduced serum insulin levels (Fig. 2A). Fasting serum glucagon-like peptide 1 (GLP-1) levels at the end of the experiment (6 months post-operative) are higher in DJOS and DiOS compared to sham (DJOS vs sham *p* = 0.0136; DiOS vs sham *p* = n.s.; DJOS vs DiOS *p* = n.s.) (Fig. 2B). Fasting serum free fatty acid (FFA) levels 6 months post-operatively are lower in DJOS and DiOS than in sham (DJOS vs sham *p* = 0.0295; DiOS vs sham *p* = n.s.; DJOS vs DiOS *p* = n.s.) (Fig. 2C).

**Fig. 3:**
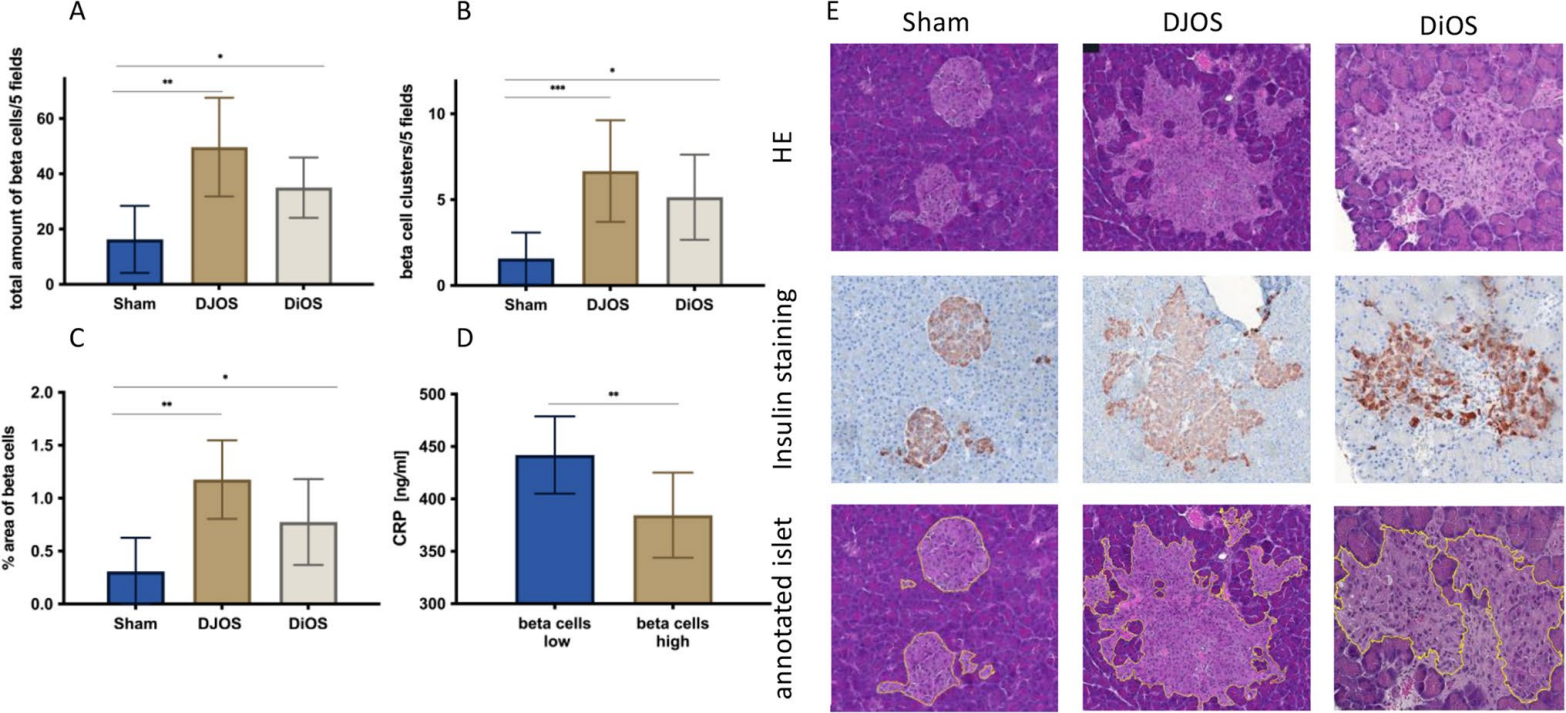
Histologic beta cell evaluation and corresponding HE and insulin stains illustrating the area of interest as defined by the AI. **A** Total number of beta cells with a preserved population of insulin-secreting beta cells in contrast to sham group (Mann–Whitney test, sham vs. DJOS *p* = 0.0023 and sham vs DiOS *p* = 0.0251). **B** Number of expanded beta cell areas in total pancreas. DJOS- and DiOS-operated animals show more expanded areas than sham animals (Mann–Whitney test, sham vs. DJOS *p* = 0.0009 and sham vs DiOS *p* = 0.0140). **C** Beta cell area in relation to total pancreatic tissue (Mann–Whitney test, sham vs DJOS *p* = 0.0012 and sham vs DiOS *p* = 0.0379). **D** Significantly lower CRP levels in animals with higher number of beta cells (Mann–Whitney test *p* = 0.0073). **E** Representative series of slides of HE and insulin immunostaining in DJOS, DiOS, and sham. Beta cells, which are somewhat difficult to recognize in HE stains, are clearly demarcated from the rest of the pancreas in the beta cell-specific insulin staining. However, the AI recognizes beta cells in the HE with high precision as can be seen in the bottom row of slides of annotated islets

### Pancreatic Beta Cells

DJOS and DiOS were associated with a significantly higher number of insulin-producing beta cells than sham (DJOS vs sham *p* = 0.0023; DiOS vs sham *p* = 0.0251; DJOS vs DiOS *p* = n.s.) and a higher number of beta cell clusters. Sham was characterized by a near-total lack of cell clusters (DJOS vs sham *p* = 0.0009; DiOS vs sham *p* = 0.0140; DJOS vs DiOS *p* = n.s.). There was a significantly lower percentage of beta cells in pancreatic tissue in sham (DJOS vs sham *p* = 0.0012; DiOS vs sham *p* = 0.0379; DJOS vs DiOS *p* = n.s.) (Fig. 3).

To correlate these findings with a systemic inflammation marker, we estimated the mean number of beta cells across all groups regardless of treatment and categorized individual mean values dichotomously as higher (8 animals) or lower (12 animals) than this value. Higher-than-mean beta cell number was associated with significantly lower levels of serum CRP levels 6 months post-operatively (beta cells low vs beta cells high *p* = 0.0073) (Fig. 3D).

### Intestinal L Cells

**Fig. 4:**
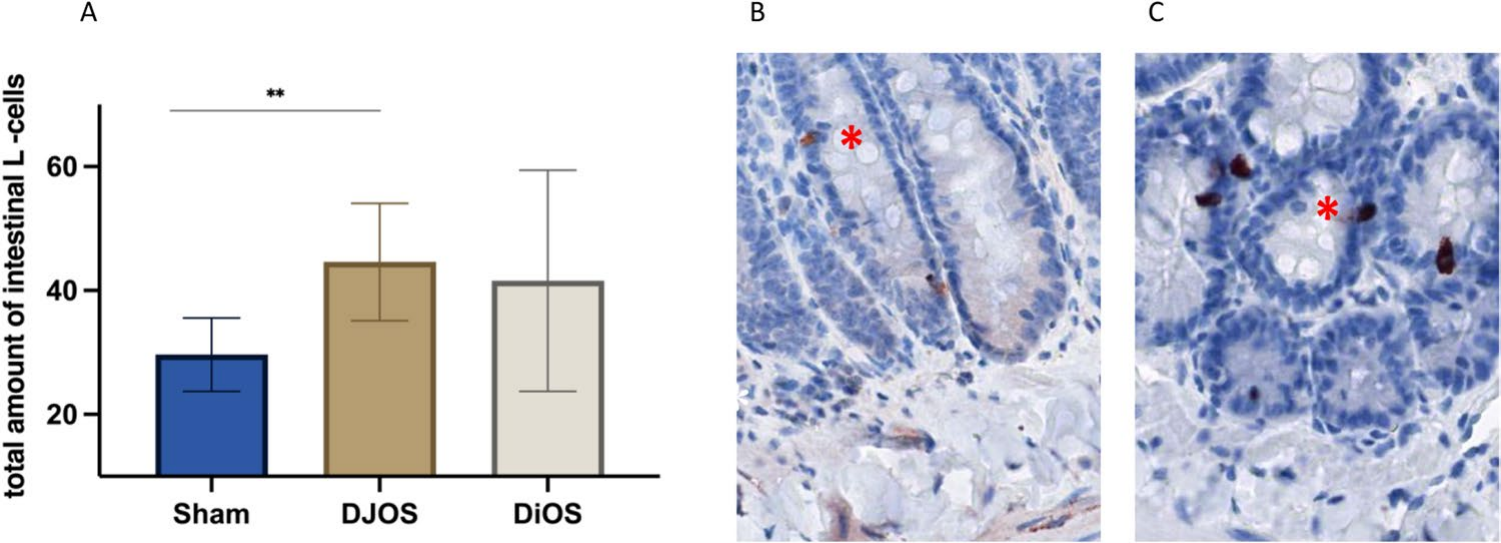
Total number of intestinal L cells in DJOS and DiOS. **A** Total L cell expression from duodenum, intestine, and ascending colon shows more GLP-1 secreting L cells in DJOS than in sham animals (Mann–Whitney test, sham vs DJOS *p* = 0.0035 and sham vs DiOS *p* = 0.2302). DJOS was significantly associated with elevated L cell number compared to sham. The difference was not significant between DiOS and Sham. **B**, **C** Representative graph of GLP-1 immunostaining in the duodenum (**B**) and the ileum (**C**) L cells stained in red, whereas enterocytes are shown in blue colored. Red asterisk shows an L cell

Increased serum GLP1 levels in the surgical groups are also reflected in increased amounts of L cells along the intestinal tract. DJOS and DiOS were associated with a higher number of L cells (DJOS vs sham *p* = 0.0035; DiOS vs sham *p* = n.s.; DJOS vs. DiOS *p* = n.s.) (Fig. 4A).

### PCNA and PDX-1

**Fig. 5:**
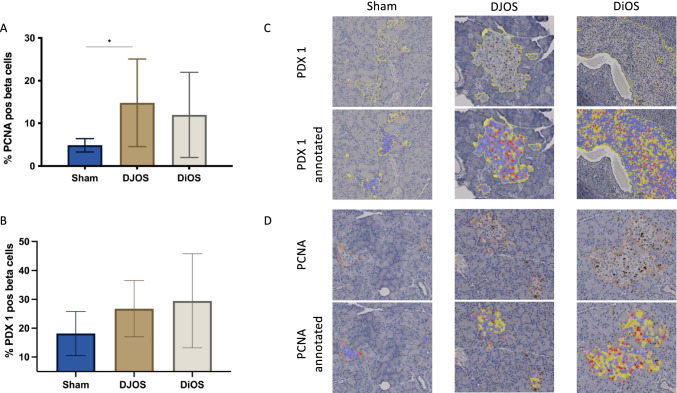
Histologic evaluation of beta cell activity with PCNA and PDX-1 immunostaining. **A** Box plot of PCNA immunostaining which shows a higher proliferation in beta cells in DJOS animals than in sham animals (Mann–Whitney test, sham vs DJOS *p* = 0.029, sham vs DiOS *p* = 0.175). **B** Box plot of PDX-1 immunostaining which shows a tendency towards higher beta cell proliferation in DJOS and DiOS. **C** Representative series of slides of PCNA immunostaining in DJOS, DiOS, and sham. Immunohistochemical staining is shown in the top row. Beta cells were very precisely labeled by the AI. In the lower panel, the evaluation performed by AI shows how strongly beta cells express PCNA. Staining intensity correlates with higher expression of PCNA. **D** Representative series of slides of PDX-1 immunostaining in DJOS, DiOS, and sham groups. Immunohistochemical staining is shown in the top row. Beta cells were very precisely labeled by the AI. In the lower panel, the evaluation performed by the AI shows how strongly beta cells express PDX-1. Staining intensity correlates with higher PDX-1 expression

PCNA served as a marker of cell proliferation. DJOS was positively associated with a significant increase of proliferation in pancreatic beta cells. This association was not as clear in DiOS (DJOS vs sham *p* = 0.0293; DiOS vs sham *p* = 0.1747; DJOS vs. DiOS *p* = n.s.). PDX-1 is a marker of early pancreatic differentiation. DJOS and DiOS were associated with a visible trend towards higher expression, but the difference did not reach statistical significance (DJOS vs sham *p* = 0.3793; DiOS vs sham *p* = 0.1851; DJOS vs. DiOS *p* = 0.6455) (Fig. 5).

## Discussion

During the natural course of disease, ZDF rats develop early and progressive depletion of beta cells and severe insulin resistance after 8 weeks post-partum. This is associated with severe, insulin-deficient diabetes in the long term [24]. We previously reported a favorable association of two types of duodenal exclusion coupled with short or long common channel with weight loss and insulin sensitivity in a ZDF model of severe genetic diabetes [22]. Both models were associated with impressive alteration of long-term diabetes progression. This data parallels high-level evidence in human studies and other rodent trial data on the efficacy of various forms of bypass surgery in rapidly improving and restoring glucoregulation [3, 25, 26].

In this study, we investigated additional metabolic and inflammatory parameters in these models and examined histopathologic findings associated with preserved insulin production. For a quantitative and reproducible IHC evaluation, an automated algorithm was applied. This type of approach has not been described for the specific purpose of examining post-bariatric pancreatic changes.

Different models are used to investigate beta cell count and morphology following bariatric surgery. Lindqvist et al. report an increase of beta cell mass in a porcine RYGB model [27]. Zhang et al. and Mosinski et al. found an increased number of beta cells in an obese rat RYGB model [28, 29]. Follow-up in these cited studies was only 4 to 8 weeks post-operatively. Wang et al. and Wu et al. demonstrate an impressive protective effect of metabolic surgery on beta cells in a streptozotocin-induced diabetes model in rats [30, 31]. Due to rapid and early depletion of beta cells during the natural course of disease in ZDF rats, this model is very suited for comparative efficacy research of bariatric or metabolic procedures. Quantitative analysis of beta cell number and area showed changes that paralleled serum fasting and dynamic insulin levels in DJOS and DiOS [22]. Increased count and larger cell clusters were characteristic in both surgical groups and contrasted sharply with sham. Sham was characterized by significantly smaller islet cell clusters and dispersed, single beta cells. These findings were corroborated by estimating the beta cell area of the entire pancreas (Fig. 3). Our follow-up of 6 months is extraordinarily long, specifically in ZDF rats. Indeed, even in this genetic model of severe diabetes, bypass surgery was associated with long-term preservation of beta cells. However, the mechanisms that underpin beta cell protection in ZDF rats are not yet sufficiently clear.

Metabolic surgery is associated with profound change of beta cell differentiation and functional state. Oppenlaender et al. investigated single-cell expression changes after bariatric surgery in a db/db mouse model [20]. Their high-dimensional analysis demonstrated rapid beta cell re-differentiation and a transition into specific functional states after metabolic surgery. However, there are contrasting reports regarding post-bariatric beta cell fate. Perez-Arana et al. [32] report on initial increase of beta cell mass with consecutive decrease below the values of control animals after 6 months in healthy, normal weight Wistar rats. They also observed reduced beta cell expression of PDX-1 and the proliferation marker Ki67 associated with RYGB. The authors observed decreased beta cell mass only in RYGB and hypothesize that beta cell transformation into alpha cells may be the underlying cause [32]. Beyond cell count and clustering patterns, we investigated features of cell function, replication, and differentiation and stained for PCNA and PDX-1 (Fig. 5). PCNA served as a marker and estimator of cell replication. Automated analysis of IHC-stained slides showed a significantly higher proportion of PCNA-positive beta cells in DJOS. In sham, almost no PCNA-positive beta cells were detectable. PDX-1 served as estimator of beta cell differentiation. Data paralleled PCNA findings and showed a visible increase of PDX-1 positivity in DJOS and DiOS compared to controls. Our data on PDX-1 and PCNA positivity in beta cells parallels the findings from Oppenlaender et al. We are not able to provide more longitudinal granularity in the molecular mechanisms of post-bariatric improvement. To what extent the observations of Perez-Arana et al. can be attributed to the non-diabetic and non-obese rat model cannot be said at present. However, in the natural course of disease, ZDF rats develop a marked decline in beta cells beginning at week 8 post-partum. By comparison, duodenal exclusion was associated with significantly improved fasting glucoregulation, increased beta cell mass, and PCNA- and PDX-1 positivity [22]. This is concordant with rodent as well as human studies of progressive severe diabetes that report beta cell de-differentiation [33, 34]. Our data are also concordant with data of Wang et al. who show an association of metabolic stress and beta cell de-differentiation as well as the re-modulation of this process through insulin therapy reduced metabolic stress [35].

The drivers of beta cell preservation, re-differentiation, and perhaps even proliferation are complex. Multiple systemic changes that involve lipotoxicity, inflammation, hyperglycemia, insulin resistance, and endoplasmatic reticulum stress are being investigated [12, 17, 36]. These aspects of metabolic improvement are inter-dependent. Defining key mechanistic drivers versus associated changes remains challenging. Here, we highlight several findings associated with post-bariatric metabolic improvement and relate them to our data.

Metabolic surgery produces a reduction and normalization of inflammatory parameters and partial to complete remission of diabetes [37]. Euguchi et al. review the association between inflammation and beta cell dysfunction [38]. They report a shift of islet macrophages towards M1 polarization in morbid obesity that are associated with inflammation and beta cell dysfunction. Wu et al. report that in an STZ model of diabetes, RYGB is associated with NLPR3 inflammasome deactivation in pancreatic macrophages and consecutive beta cell function improvement [31]. In our model, elevated CRP was associated with significantly fewer islet cells (Fig. 3D).

GLP-1 is associated with reduced inflammation and beta cell function. Eizirik et al. and Izaguirre et al. report an association of increased GLP-1 with improved insulin sensitivity and reduced inflammation [11, 39]. In fact, low preoperative levels of GLP-1 were predictive of poor outcome after RYGB surgery. Also, parenteral GLP-1 administration significantly reduces systemic inflammation and improves peripheral insulin sensitivity [39]. Is GLP-1 the main etiologic factor of diabetes improvement? Our data and data of other groups also show a significant association between improved peripheral insulin sensitivity, increased GLP-1 levels, and pancreatic function [40–42]. Chronic hyperglycemia leads to beta cell ER stress and results in cellular dysfunction and apoptosis [43]. ER stress in beta cells is associated with development of diabetes in rodent and human studies [44–47]. GLP-1 reduces beta cell ER stress and promotes beta cell proliferation and function via multiple pathways [12, 48–50].

ZDF rats also develop extreme lipotoxicity and extreme levels of free fatty acids (FFA), triglycerides, and cholesterol [24, 51, 52]. Lipotoxicity has been associated with beta cell loss and insulin resistance [53]. Additionally, Li et al. recently reported on GLP1-mediated protection of beta cells mediated of lipotoxic effects via a PARP-pathway [54]. Concordantly, we observed an improvement of lipid metabolism after bypass surgery.

From a clinical point of view, each of these findings demonstrates important aspects of post-bariatric metabolic improvement in DiOS and DJOS. The complex relations between these molecular mechanisms remain controversial areas of research.

In summary, this model of foregut exclusion testing two different common channel lengths shows impressive alteration of the natural course of diabetes progression and strong, long-term metabolic effects in ZDF rats. Translating these findings to the clinical context, it appears relevant that common channel length is not associated with systemic or histologic parameters in this bypass model of severe diabetes. Mechanistically, it remains unclear whether GLP-1 is the main driver of beta cell protection and function as well as reduced inflammation. Our data demonstrate that metabolic surgery, independently of common channel length, induces massive and very long-term histomorphologic modulation of the pancreas and specifically of beta cells regardless of common channel length. In conclusion, long-term metabolic improvements characterized by improved weight trajectories, hormonal levels, and parameters of fasting glucoregulation and insulin sensitivity are associated with increased beta cell count and altered morphology as well as histologic features of proliferation and differentiation in a ZDF model. We leveraged an artificial intelligence algorithm approach to investigate these changes.
